# Recent Progress of Energy-Storage-Device-Integrated Sensing Systems

**DOI:** 10.3390/nano13040645

**Published:** 2023-02-06

**Authors:** Man Yuan, Xinqun Zhang, Jiaqi Wang, Yang Zhao

**Affiliations:** Key Laboratory of Cluster Science, Ministry of Education of China, Key Laboratory of Photoelectronic/Electrophotonic Conversion Materials, School of Chemistry and Chemical Engineering, Beijing Institute of Technology, Beijing 100081, China

**Keywords:** sensing, energy storage, integrated system

## Abstract

With the rapid prosperity of the Internet of things, intelligent human–machine interaction and health monitoring are becoming the focus of attention. Wireless sensing systems, especially self-powered sensing systems that can work continuously and sustainably for a long time without an external power supply have been successfully explored and developed. Yet, the system integrated by energy-harvester needs to be exposed to a specific energy source to drive the work, which provides limited application scenarios, low stability, and poor continuity. Integrating the energy storage unit and sensing unit into a single system may provide efficient ways to solve these above problems, promoting potential applications in portable and wearable electronics. In this review, we focus on recent advances in energy-storage-device-integrated sensing systems for wearable electronics, including tactile sensors, temperature sensors, chemical and biological sensors, and multifunctional sensing systems, because of their universal utilization in the next generation of smart personal electronics. Finally, the future perspectives of energy-storage-device-integrated sensing systems are discussed.

## 1. Introduction

With the mature development of electronic technology, the demand for smart sensing systems is increasing rapidly, especially toward real-time wireless monitoring of changes in the human body and environment by smartphones or watches [1,2,3,4]. In past decades, numerous sensors that detect various physical and chemical information have been widely developed [5] including tactile sensors [6,7,8,9], temperature, sensors [10,11,12], image sensors [2], humidity sensors [13], and chemical and biological sensors [14,15,16]. These well-developed sensors show the advantages of thinness, a small volume, light weight, and flexibility, and provide promising platforms for flexible and portable intelligent sensing systems. However, they still need an external power supply, which greatly limits their practical application.

**Figure 1:**
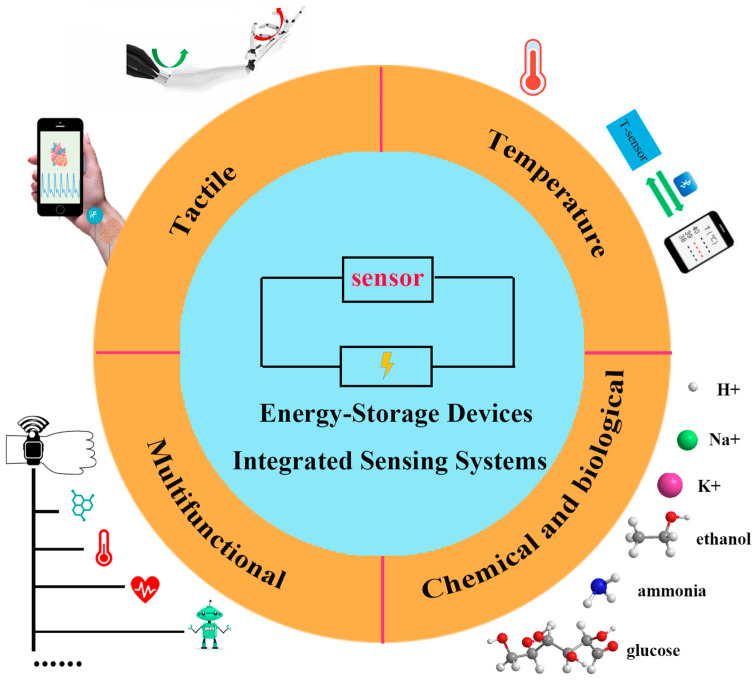
Schematic diagram of Energy-storage-device-integrated Sensing Systems. Reprinted with permission from Ref. [33]. Copyright 2021, Advanced Materials. Reprinted with permission from Ref. [34]. Copyright 2020, Science Robotics. Reprinted with permission from Ref. [35]. Copyright 2020, Biosensors and Electronics.

To solve the above problem, self-powered sensing systems without external power supplies, including energy-harvester-integrated systems and energy-storage-device-integrated systems, are regarded as effective methods and have received great attention. In energy-harvester-integrated systems, various forms of energy can be converted into electrical energy in a specific way to drive the sensors, such as the triboelectric and piezoelectric effects for mechanical energy [17,18], the photovoltaic effect for solar energy [19], and the thermoelectric and pyroelectric effects for thermal energy [20]. However, the energy-harvesters usually need to be exposed to specific energy sources to work, so their application scenarios are greatly limited, and the low energy conversion rate leads to poor stability and continuity. In contrast, sensing systems integrated with energy-storage devices can greatly avoid these drawbacks, and will work directly and effectively. Generally speaking, energy-storage devices come in a variety of types, such as batteries (lithium-based batteries [21,22,23,24], zinc-based batteries [25,26], and emerging biofuel cells [27]), supercapacitors [28,29,30], and hybrid devices [31,32]. In recent years, the flexible energy-storage devices that are compatible with sensor components have been developed with an increasingly mature manufacturing process, which provides more possibilities for wearable electronics in practical meaning. Energy-storage-device-integrated sensing systems further connected with the energy-harvesters, especially, will dominate the main trend of wearable and flexible electronics in the future [2,4,27]. In the past, there were some overviews on self-powered sensing systems, and the energy-storage devices integrated sensing systems were briefly described as a small part of them, but few overviews focused on them. Therefore, an overview of the energy-storage-device-integrated sensing systems is provided here. We summarize the recent achievements of four main types of energy-storage-device-integrated sensing systems, including tactile, temperature, chemical and biological, and multifunctional types, considering their irreplaceable position in the fields of human health monitoring, intelligent robots, human–machine interaction, and so on (Figure 1). At last, the challenges and perspectives of wearable electronics are also discussed.

## 2. Energy-Storage-Device-Integrated Sensing Systems

Table 1 summarizes the characteristics of energy-storage devices and integration modes for various systems in this review. Next, we will introduce different types of energy-storage-device-integrated sensing systems from the functional perspective, and summarize their advantages and disadvantages, as well as future optimization direction in this part.

### 2.1. Tactile Type

Tactile sensors convert tactile changes (friction, pressure, stress, etc.) into electrical signal changes through certain principles. According to the transduction mechanisms, they can be divided into resistive, capacitive, piezoelectric, and triboelectric types. In recent years, the materials, manufacturing approaches, performance optimization strategies of sensors, and their applications in the fields of human–machine interaction, electronic skin, and personal health monitoring have been widely studied [36,37,38,39]. In this section, we will cover the recent progress of energy-storage-device-integrated tactile sensing systems according to the transduction mechanisms.

Resistive Tactile Sensors: Resistive tactile sensors convert the applied tactile input into resistance variation based on energy band change of semiconductor or percolation theory, tunneling effect, or interface contact resistance change of conductor, which are widely used, due to their simple manufacturing process, high sensitivity, stable performance, and wide detection range [37,40]. Various conductive materials are usually applied in the resistive tactile sensors, such as carbon materials (carbon nanotubes (CNTs), graphene, carbon black, etc.), conductive polymers, low-dimensional metallized nanomaterials, and so on, which are often compounded with elastomers (polydimethylsiloxane (PDMS), polyurethane (PU), etc.), followed by microstructure engineering to improve performance [36,37,38,39,40,41].

**Figure 2:**
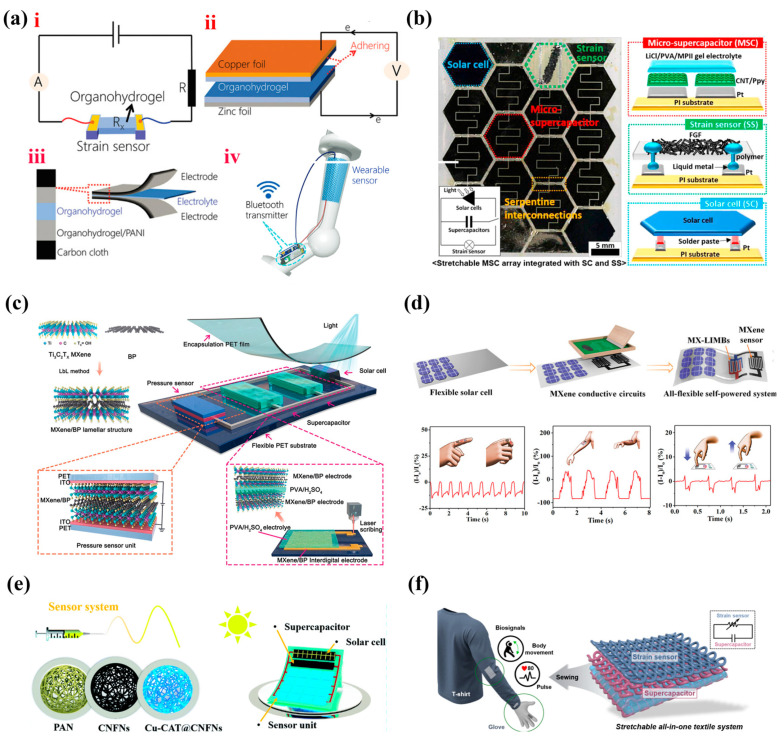
(**a**) Illustration of i. strain sensor, ii. battery, iii. Supercapacitor, and iv. wireless wearable sensor prototypes. Reprinted with permission from Ref. [42]. Copyright 2022, Advanced Functional Materials. (**b**) Optical image and schematic illustration of the biaxially stretchable integrated system. Reprinted with permission from Ref. [47]. Copyright 2018, Nano Energy. (**c**) Schematic illustration of the MXene/BP-based integrated sensing system. Reprinted with permission from Ref. [48]. Copyright 2021, Advanced Materials. (**d**) Schematic diagram of integrated system prepared by printing techniques and its current change in response to the bending of a finger, the bending of an elbow, and pressing vertically. Reprinted with permission from Ref. [53]. Copyright 2021, Advanced Materials. (**e**) Manufacturing strategy of the all-fiber supercapacitor-integrated pressure sensing system. Reprinted with permission from Ref. [55]. Copyright 2022, Journal of Materials Chemistry A. (**f**) Structure of textile-integrated system and conceptual diagram sewn onto clothes. Reprinted with permission from Ref. [63]. Copyright 2019, ACS Nano.

To realize the integration of resistive tactile sensors and energy-storage devices, the types of materials and the component manufacturing methods need to be considered. In the early stage, researchers have tried to explore and develop multipurpose materials that can be used in both the tactile sensing and energy storage fields. In fact, many kinds of materials can be used for both tactile sensing and energy storage due to their excellent conductivity and special microstructure, including hydrogel [42,43], polypyrrole/β-FeOOH/nylon strip [44], reduced graphene oxide/polyaniline wrapped carbonized sponge [45], phosphorene-incorporated flexible 3D porous graphene [46], and so on. For example, Wu et al. [42] developed a kind of organohydrogel with adhesiveness and a high robustness for strain sensor (Figure 2a(i)), battery (Figure 2a(ii)), and supercapacitor (Figure 2a(iii)) manufacturing. For the strain sensor, the tensile strain caused changes in the cross-sectional area and length of the organohydrogel, and thus induced a resistance change. By combination of the organohydrogel and a commercial Bluetooth transmitter (Figure 2a(iv)), it could be used to wirelessly measure motions with high sensitivity (gauge factor: 8.82), a fast response, and excellent reversibility. After introducing Cu^2+^ and Zn^2+^ into the organohydrogel, it could be directly used as the electrolyte of the battery. The as-assembled battery exhibited an open circuit voltage of 1.02 V and remained stable for 144 h. Additionally, the organohydrogel compounded polyaniline was used to prepare a sandwich supercapacitor, showing a capacitance of 14.3 mF cm^−2^. However, the research of these above studied materials in energy-storage devices and sensors were separate and independent, so there is a need to achieve integration of functions (or devices) to approach the actual application.

Later, an integrated system containing a solar cell as the energy-harvester, an array of micro-supercapacitors, and a strain sensor was successfully developed by Ha et al. [47] on stretchable substrate (Figure 2b), which realized real-time pulse monitoring and bending motions of human joints. It can efficiently avoid the cumbersome external circuit in Figure 2a(iv). Unfortunately, the tedious manufacturing process (especially the manufacture of an array of supercapacitors) greatly limited its practical application. Another simple study for the integrated system was carried out by Han et al. [48] by using periodic interleaving MXene/black phosphorus (MXene/BP) multilayer films (Figure 2c). The periodic insertion of BP expanded the layer spacing of MXene flakes, which accelerated ion transport, provided facile access to Ti atoms, and resulted in a high deformability, which not only provided high electrochemical activity for the micro-supercapacitor, but also realized various human signal monitoring (limb bending and pulse) as a pressure sensor. The resultant integrated system exhibited a high energy-storage capacity (specific capacitance: 896.87 F cm^–3^ at 0.69 A cm^–3^) and an excellent pressure sensing performance (sensitivity: 77.61 kPa^−1^; response time: 10.9 ms). However, the preparation of MXene/BP through periodic alternative filtration of MXene and BP was not conducive to large-scale production. In recent years, the printing techniques applicable to various flexible substrates have attracted extensive attention, and provided alternatives for manufacturing various flexible electronic components and integrated systems [49,50,51,52]. For example, Wu et al. developed printable multitasking MXene inks for current collector, energy storage, and sensing materials [53]. The MXene inks were directly used in supercapacitor manufacturing, which exhibited an ultrahigh areal capacitance of 1.1 F cm^−2^. The MXene-based lithium titanate and lithium iron phosphate inks were used in micro-battery manufacturing, and offered an excellent areal energy density of 154 μWh cm^−2^. A pressure sensor using MXene ink–polyvinyl alcohol hydrogel as piezoresistive layer was integrated with a micro-battery and a solar cell to realize self-powered pressure sensing (Figure 2d). However, the sensor, composed of a piezoresistive layer without microstructure and inter-digitated electrodes [54], has a relatively weak sensing performance compared to those with microstructures. In addition, breathability and comfort are equally important for wearable electronics. Wang et al. [55] manufactured a pressure sensing system with a coplanar integrated structure similar to the above by utilizing an all-fiber structure covered with two-dimensional conductive MOF materials (Figure 2e). Benefiting from the shell-core structure of active materials and all-fiber structure of devices, this system presented high sensitivity (30 kPa^−1^), outstanding capacitance (264.8 mF cm^−2^), good air permeability, and scalability. In addition, the integrated devices built on the fabrics would have better breathability and comfort than other common flexible substrates, and there are precedents for building energy-storage devices [56,57,58,59] and tactile sensors [59,60,61,62] on our daily fabrics. For example, an all-fabric integrated system with a strain sensor and a supercapacitor has been developed (Figure 2f) [63], in which MWCNT/MnO_3_ and the three-dimensional structure of fabrics endowed a supercapacitor with excellent energy storage and mechanical properties, as well as the strain sensor with excellent sensitivity and cycling performance.

Capacitive Tactile Sensors: The working principle of capacitive tactile sensors mainly depends on the applied tactile input to change the distance between electrodes or the electrode area, resulting in capacitance variation. Compared to the resistive type, capacitive sensors consume less energy and are insensitive to temperature and humidity, but exhibit relatively low linear dynamic range and are sensitive to electromagnetic interference [64]. Appropriate electrode materials and dielectric layer materials are essential for capacitive sensors, and the introduction of microstructure can further improve sensing performance [38,64,65]. In addition, the field effect transistor (FET) structure can self-amplify the piezo-capacitive response [66]. Capacitive tactile sensors are inherently capable of storing energy, but it is difficult for a single device to perform well in sensing and energy storage at the same time. These dual-function capacitors are relatively few, the key is to find a balance.

**Figure 3:**
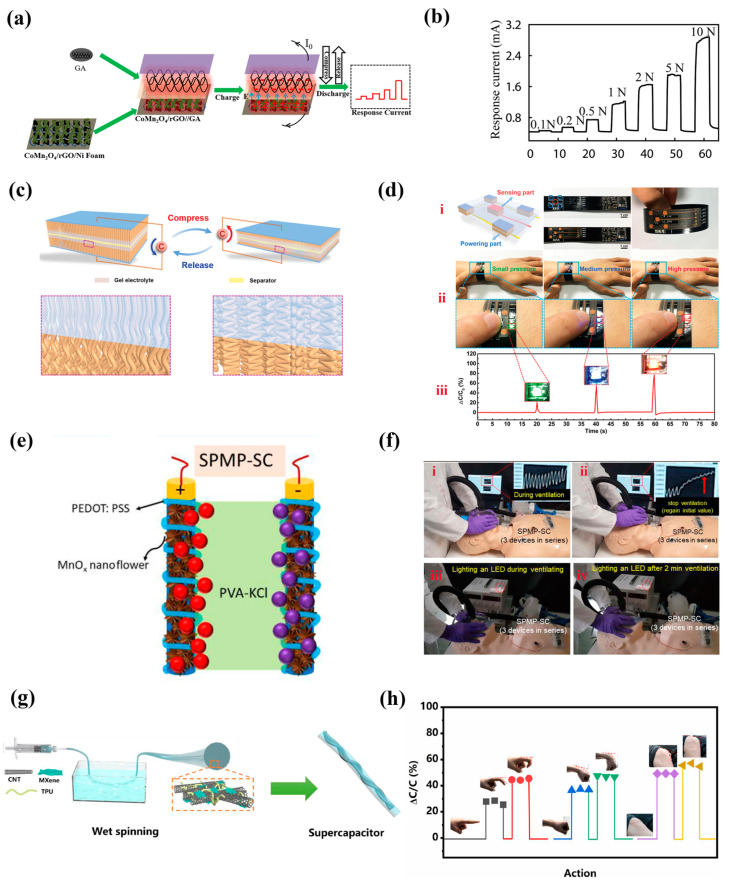
(**a**) Schematic illustration and application of GA-based supercapacitors and (**b**) its I–t curves at different pressures ranging from 0.1 to 10 N. Reprinted with permission from Ref. [70]. Copyright 2018, Journal of Power Sources. (**c**) CCNAs-based supercapacitors function as a strain sensor before and after compressing. (**d**) i. Schematic diagram and photographs of the integration of CCNAs-based supercapacitors. ii. Different degrees of pressure being applied on the integrated system to light up LED. iii. Relative capacitance responses obtained from the CCNAs-based supercapacitors during the compressing process. Reprinted with permission from Ref. [74]. Copyright 2020, Advanced of Materials. (**e**) Schematic illustration of SPMP-SCs. (**f**) Dual function of the SPMP-SC (i, ii) as an intrinsic strain sensor for monitoring the chest expansion during ventilation. (iii, iv) Constant power out of the SC during ventilation (stretching) by lighting an LED. Reprinted with permission from Ref. [75]. Copyright 2021, ACS Applied Materials & Interfaces. (**g**) Preparation and (**h**) Capacitance changes corresponding to actions of fibrous supercapacitors. Reprinted with permission from Ref. [76]. Copyright 2021, Electrochimica Acta.

The abundant pore structures of graphene aerogel (GA) is conducive to the entry of electrolytes and can realize multi-dimensional electron transport, which is widely used in capacitor energy storage [67,68,69]. It is also applied as a resistive tactile sensor due to its excellent elasticity [6,70,71]. Based on GA material, Zhao et al. [70] reported an elastic supercapacitor using CoMn_2_O_4_/rGO as the positive electrode and N, B, and S co-doped elastic GA as the negative electrode, which exhibited a high energy density (53.33 W h Kg^−1^) at a power density of 400 W Kg^−1^ (Figure 3a). The elastic GA endowed the supercapacitor with pressure sensing performance (0.1–10 N). The applied pressure caused the change of an internal electric field, which was further converted into self-discharge current variation (Figure 3b). Oriented CNTs arrays are another excellent energy storage and tactile sensing material [72,73]. A kind of compressible CNT array (CCNA) with a gradually crosslinking structure was reported by Peng et al. [74] (Figure 3c), which exhibited highly reversible compressibility of more than 100,000 cycles. A single supercapacitor based on CCNA could function as both an energy storage device and pressure sensor; the capacitance changed steadily with the electrode thickness when external pressure was applied. An integrated device consisting of four supercapacitors in series as power supply and a middle capacitive sensing unit is shown in Figure 3d i, which could effectively sense different pressure changes. However, the harsh preparation condition of CCNA limited its application. To avoid the weak and poor mechanical properties (especially tensile deformation) by GA- and CCNA-based devices, fibers as structural support for loading active materials have attracted great interest and proved to be effective. For example, Dahiya et al. [75] used Lycra fabric as a substrate for PEDOT:PSS/MnO_x_/PEDOT:PSS-based supercapacitor fabrication (SPMP-SCs, Figure 3e). The SPMP-SCs encapsulated by Ecoflex realized strain monitoring based on the disconnection mechanism with a sensitivity of −0.4% and a high capacitance retention above 90% after 1000 cycles of 40% stretching. As shown in Figure 3f, an SPMP-SC was used for capturing the volumetric expansion of the mannequin’s chest, and could also stably supply energy for an LED during this period. In addition, fiber materials are also suitable for the manufacture of one-dimensional stretchable fibrous supercapacitors that are more flexible and easier to integrate with clothes. A kind of stretchable fibrous supercapacitor based on CNTs/MXene-TPU hybrid fibers was reported by Wang et al. [76] (Figure 3g), which exhibited high energy density (1.16 mWh cm^−3^) comparable to the commercial supercapacitors due to the addition of MXene and the porous structure of hybrid fibers. Meanwhile, it also possessed strain sensing performance because of introduction of a TPU elastomer (Figure 3h). Thus, it can be seen that fibrous substrates have great potential in stretchable dual-function supercapacitors manufacturing due to the advantages of porosity, good ductility, low cost, and easy functionalization.

It is not difficult to see that compared with traditional capacitive sensors [7,77,78,79], the sensing performance of these dual-function capacitors is relatively poor. Most of them can only monitor strong signals such as bending and stretching, and can do nothing for tiny physiological signals such as pulse.

Piezoelectric Tactile Sensors: Piezoelectric materials can produce a polarization charge proportional to the applied pressure on the surface; this unique energy transduction enables their applications in fields of energy harvesting and tactile sensing [64,80]. Many inorganic and organic polymer piezoelectric materials, such as ZnO [81], lead zirconate titanate (PZT) [7,18], and polyvinylidene fluoride (PVDF) [81,82] have been used for self-powered tactile sensors with high sensitivity and fast response time. Most sensors based on the piezoelectric effect can only be used for dynamic pressure measurement. In addition, owing to their unstable output power, external energy storage systems are still required to realize wireless sensing.

**Figure 4:**
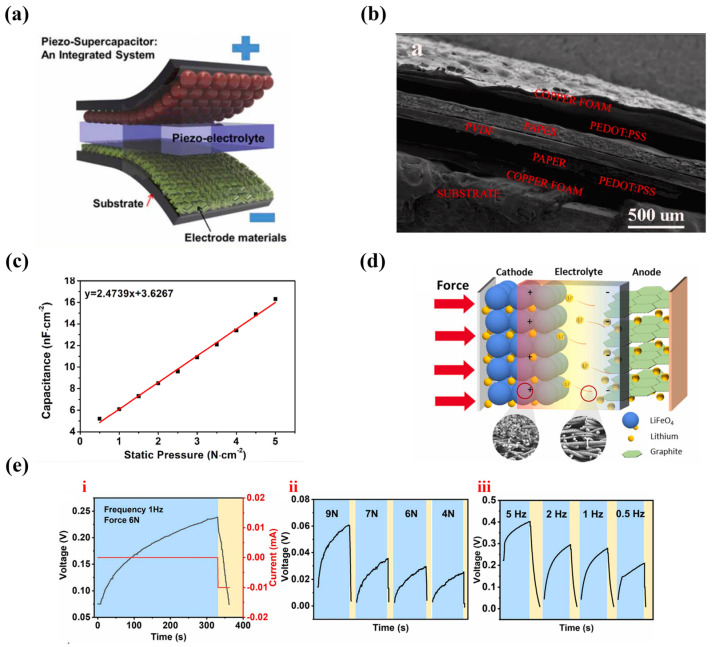
(**a**) Schematic of the PSCs. Reprinted with permission from Ref. [83]. Copyright 2021, Nano Energy. (**b**) SEM diagram of the tactile sensors based on PSCs. (**c**) Relationship between capacitance and externally applied static force. Reprinted with permission from Ref. [84]. Copyright 2019, Nano Energy. (**d**) Schematic of the SCPCs. The self-charging performance of flexible SCPC: (**e**) i. periodic external force; ii. different force strengths; iii. force frequencies. Reprinted with permission from Ref. [85]. Copyright 2022, Nano Energy.

Therefore, piezoelectric sensors integrated with energy storage systems are extremely beneficial for their practical application. As shown in Figure 4a [83], coupling piezoelectric materials into supercapacitors to form piezoelectric supercapacitors (PSCs) can not only harvest and store energy, but also realize pressure monitoring. The polarization charge generated by piezoelectric material produces potential difference between the positive and negative electrodes, driving the anions and cations in the electrolyte to move to a new balance, which is similar to the charging process of a supercapacitor, so as to realize energy storage. PSCs-based tactile sensors with PVDF as piezoelectric materials, paper sheets soaked with PEDOT: PSS as positive and negative electrodes were developed by Cao et al. [84] as shown in Figure 4b, which simultaneously realized static/dynamic pressure monitoring. The developed PSC-based sensor could charge itself up to 150 mV per unit in 300 s by tapping. Its static detection ranged from 0.5 to 5 N·cm^−2^ with a sensitivity of 2.47 nF N cm^−2^ and a detection limit of 0.1 N·cm^−2^ (Figure 4c), while dynamic detection ranged from 1 to 5 N·cm^−2^ with a sensitivity of 15.5 mV cm^2^·N^−1^ and a detection limit of 0.4 N·cm^−2^. In addition, storing mechanical energy in batteries has also proven to be effective. A novel flexible self-charging power cell (SCPC) was prepared based on electrospinning fluoride-trifluoro ethylene (P(VDF-TrFE)) porous membranes as the piezoelectric separator and supporting layer of the electrode [85] (Figure 4d). The SCPC sealed in a flexible case could harvest and store the tiny movement energy of human body under low frequency and low pressure, which charged itself up to a storage capacity of 0.092 μA h in 330 s by compression (6 N,1 HZ) (Figure 4e(i)). The maximum charging voltage was positively correlated with pressure and frequency (Figure 4e(ii,iii)). This type of energy harvest-storage system based on the piezoelectric effect has been widely studied [83]. Although most of them can realize sensing, energy harvest, and storage, their sensing performance (sensitivity, detection range and limit, response time) is relatively low compared to traditional piezoelectric pressure sensors.

Triboelectric tactile sensors: Triboelectric nanogenerators (TNGs) are also extensively used in self-powered tactile sensing systems by harvesting mechanical energy since the first prototype was reported in 2012 [17,86,87]. For instance, Chen et al. [33] developed a textile triboelectric sensor and further integrated it with a Bluetooth transmission module, realizing real-time wireless external pressure monitoring through the cellphone application. TNGs-based electronic skin [88], fiber [89], visualized flexible film [90], and smart gloves [91] have also been developed. Despite this, TNGs have the defect of unstable output power, so it will be effective to integrate them with an energy storage system.

**Figure 5:**
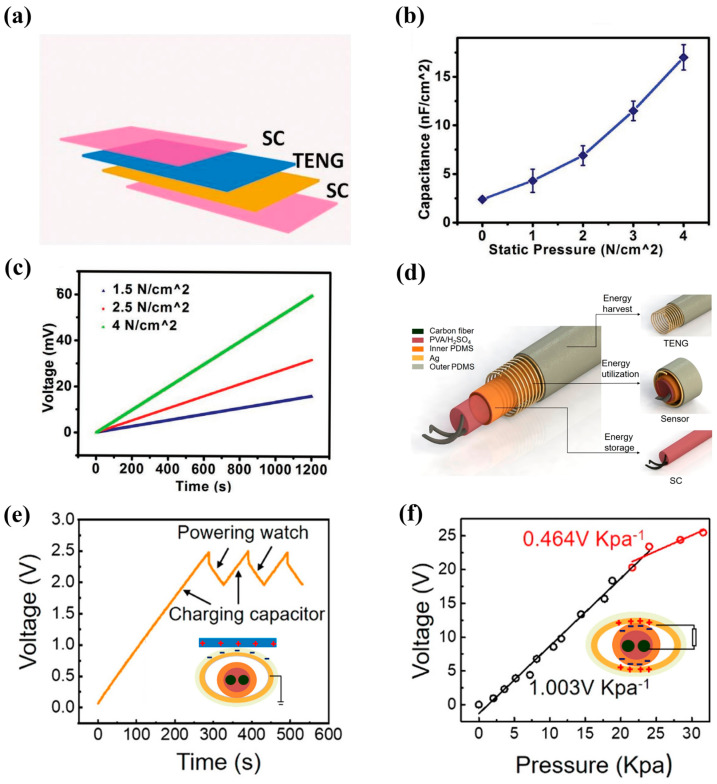
(**a**) Schematic illustration of TSC-based pressure sensor and its (**b**) capacitance–static pressure curve; (**c**) voltage under periodic external force of the same frequency (2 Hz). Reprinted with permission from Ref. [92] Copyright 2018, Advanced Energy Materials. (**d**) Schematic structure diagram of the multifunctional coaxial energy fiber. (**e**) Voltage–time curve of capacitor during charging of the capacitor and powering an electronic watch. Insert is the schematic diagram of single-electrode mode (**f**) Force–voltage curve of the integrated fiber, and the insert shows the contact-separation mode. Reprinted with permission from Ref. [93]. Copyright 2021, ACS nano.

In addition to being directly connected to energy-storage devices, triboelectric materials can also be coupled into supercapacitors like PSCs. A triboelectric supercapacitor (TSC)-based pressure sensor was developed by Wang et al. [92] (Figure 5a), which enabled the detection of both static and dynamic pressures, Figure 5b shows the relationship of capacitance and static pressure. Figure 5c illustrates the self-charging performance of a TSC under different periodic external forces (2 HZ). TSCs in series could supply power for electronic products such as LEDs and electronic watches, and their voltage is kept constant for a long time in the case of an open circuit. Wang et al. [93] also reported another uncoupled and highly integrated multifunctional coaxial energy fiber that consists of a fiber supercapacitor and a TNG fiber, as shown in Figure 5d. The fiber supercapacitor presented a good specific length capacitance density of 13.42 mF cm^−1^ and remained unchanged after 1000 cycles of bending, which proved its excellent bendability. This TNG fiber provided a maximum power of 2.5 μW in single-electrode mode, realizing continuous charging for capacitors to drive the watch or temperature and humidity sensor (Figure 5e). In the contact-separation mode, it exhibited a pressure sensing sensitivity of 1.003 V·kPa^−1^ and was used for motion monitoring and tactile interface (Figure 5f). Unfortunately, TNG fiber power for the fiber supercapacitor has not been shown in this study. We suspect that the power of the TNG was too small and can only supply power for small capacity capacitors (10, 33, 100 uF).

Although most energy-harvester-integrated systems based on piezoelectric and triboelectric materials have realized continuous wireless monitoring, the energy conversion efficiency is still low and unstable. Therefore, the utilization of energy-storage devices is extremely necessary. In addition, the stored electric energy can also be used to drive other sensors, paving the way for multi-sensing function integrated systems.

### 2.2. Temperature Type

There is a huge demand for sensors that can stably and continuously measure human body or ambient temperature. The traditional rigid temperature detectors can only meet some of the needs because of inflexibility, poor comfort, and low portability. Basically, there are four main types of flexible temperature sensors: resistance temperature detectors, thermocouple sensors, thermistor sensors, and thermochromic sensors [94,95,96]. Most self-powered temperature sensors are manufactured according to the Seebeck effect, which converts temperature into voltage signals and can be used for sensing and energy harvesting [97,98,99,100]. In addition, the temperature sensors can be powered by harvesting other forms of energy. However, these types powered by energy-harvesters are limited to specific application scenarios, and energy-storage integrated temperature sensors can provide a wider range of applications. Therefore, several typical studies of the energy-storage-device-integrated temperature sensors will be shown in the following section.

**Figure 6:**
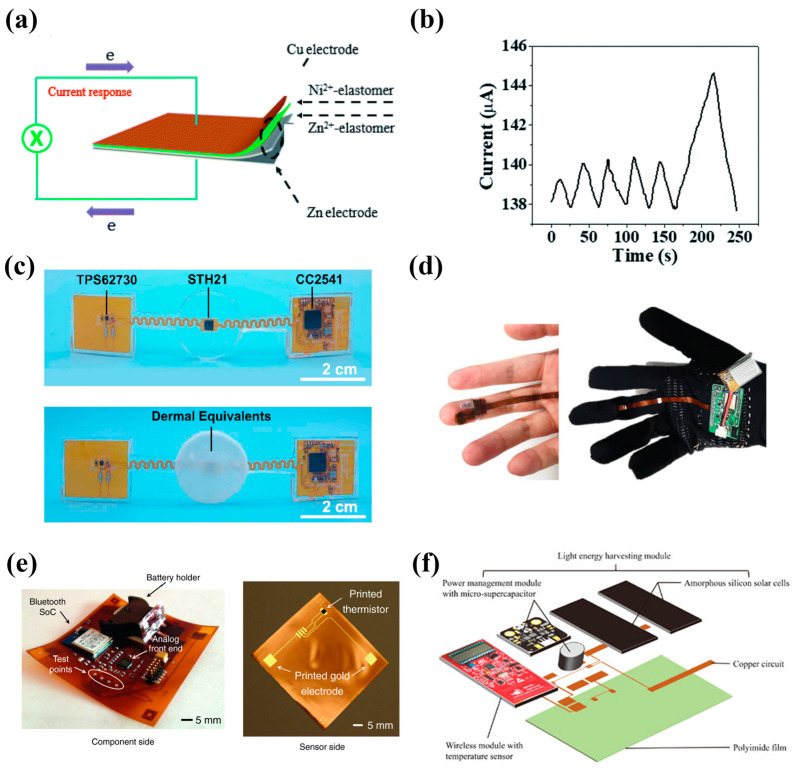
(**a**) Schematic diagram of the galvanic cell. (**b**) The current change of contactless temperature-detection. Reprinted with permission from Ref. [106]. Copyright 2022, Journal of Materials Chemistry A. (**c**) Schematic of the wireless sensing system for wound temperature monitoring. Reprinted with permission from Ref. [35]. Copyright 2020, Biosensors and Bioelectronics (**d**) Photograph of the integrated system for skin moisture and temperature wireless monitoring. Reprinted with permission from Ref. [107]. Copyright 2021, Advanced Healthcare Materials. (**e**) Photograph of the component side and the sensor side (before component assembly) of the patch. Reprinted with permission from Ref. [108]. Copyright 2016, Advanced Functional Materials. (**f**) Illustration of structural formation of LTSP. Reprinted with permission from Ref. [109]. Copyright 2021, ACS Applied Electronic Materials.

In addition to the simple integration of temperature sensor and energy storage unit [101,102,103,104], some energy storage units can realize temperature sensing themselves [105,106]. For example, a Cu-Zn galvanic cell with both Ni^2+^-containing elastomers and Zn^2+^-containing elastomers as solid electrolyte layers was developed by Wang [106] (Figure 6a), which exhibited excellent temperature sensing performance in the range of 25–60 °C according to the Arrhenius relationship between current density and temperature. As shown in Figure 6b, it could be used to detect a temperature change of 0.3 °C by the palm contactless approach. In addition, it could also be used as a pressure sensor due to the elasticity of the electrolyte layer. Even though the studies mentioned above require external equipment to collect and process data, which is far from the practical application of portable intelligent temperature sensors, it is of great significance to develop and explore efficient ways for the integration of data processing and transmission devices. As shown in Figure 6c, a sensing system containing Bluetooth wireless signal transmission was developed for wound temperature monitoring by Ma et al. [35]. Electronic components and a temperature sensor were designed on the upper of the patch, and the lower layer was collagen–chitosan dermal equivalent for skin regeneration. This patch featured flexibility, high accuracy (deviation < 0.1 °C), reliability, and biocompatibility, but the only deficiency was that there was no integrated and adaptive energy storage system.

In a separate study, a system for skin moisture and temperature wireless monitoring was reported and shown in Figure 6d [107], in which the humidity and temperature sensing layers were graphene/ZnIn_2_S_4_ and carbon CNT/SnO_2_, respectively. Although they have designed a special jack to connect with the lithium battery and achieved wearability, the huge volume and inflexibility of the energy storage device, data processing, and transmission circuit greatly reduced its wearing comfort. In an earlier study, Arias et al. [108] integrated various hard components that included a battery holder on one side of a single Kapton polyimide patch, printed gold electrodes for electrocardiography (ECG) sensing, and a printed NiO thermistor for temperature sensing on the other side, as shown in Figure 6e. The thermistor exhibited a temperature coefficient of −5.84% K^−1^, a material constant of 4330 K, and the sensing range covered the temperature range of human skin (32–37 °C). This system was powered by a commercial button battery, which would involve battery replacement and may cause environmental pollution and inconvenience. In this regard, the intense integration of a rechargeable battery and flexible substrate will be very promising. It is worth noting that the fabrication method of integrating hard and flexible electrons in this system can be extended to other wearable sensing fields. Similarly, a light-energy-harvested flexible wireless temperature-sensing patch (LTSP) for food cold storage was manufactured on a polyimide film, and a liquid crystal display was also engaged for the display of temperature-sensing data and micro-supercapacitor voltage [109] (Figure 6f). The light energy was harvest by the solar cells and stored in a commercial micro-supercapacitor (10 mF) through the energy management module, then supplied power for the commercial temperature sensor and electronic components. In addition, data could be read through near-field communication (NFC) or radiofrequency identification (RFID). Systems that output data in multiple ways like this may further improve the usability of wearable electronics. Unfortunately, LTSP was not suitable for human body temperature measurement due to its large volume and low flexibility. Generally, the energy-storage-device-integrated sensing systems used for human body detection should have excellent resolution, and sometimes need to fit closely with human skin, which puts forward higher requirements for the safety, flexibility, long-term stability, and comfort of sensing and energy storage materials.

### 2.3. Chemical and Biological Type

As people pay more attention to health, traditional diagnostic instruments are bulky and expensive, which cannot meet the needs of personalized healthcare, telemedicine, and early disease diagnosis in the future. Wearable chemical and biological sensors for different kinds of biological and environmental indexes have been extensively applied [110,111]. Although the use of energy-harvesters for power supply [112], wireless coil power supply [113], and colorimetric analysis [114,115] has been proven to be effective, the chemical and biological sensing systems with energy-storage devices facilitate wireless data transmission and collection, which are essential for determination and alarm of dangerous chemical substances and prevention, diagnosis, and treatment of diseases. In this part, we mainly summarize some energy-storage-device-integrated sensing systems used for harmful gas monitoring and biochemical markers.

**Figure 7:**
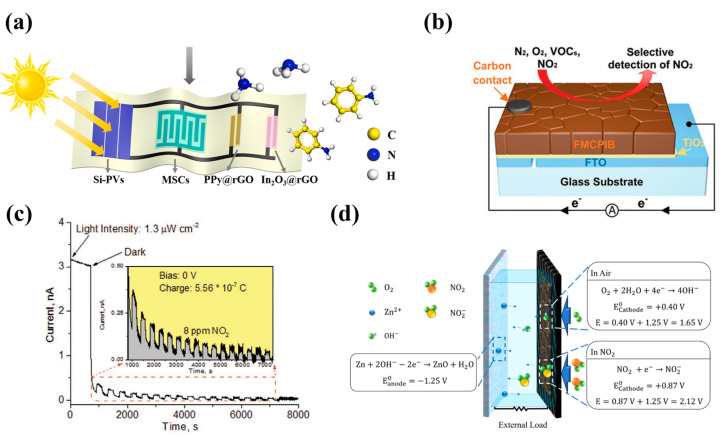
(**a**) Schematic of seamlessly integrated solar cell, micro-supercapacitor, and gas sensor on a flexible substrate. Reprinted with permission from Ref. [119]. Copyright 2021, Nano energy. (**b**) The schematic of perovskite-based device and its (**c**) dynamic current response under irradiation and darkness. Reprinted with permission from Ref. [126]. Copyright 2020, Advanced Optical Materials. (**d**) Key reactions and mechanisms of battery–sensor hybrid device. Reprinted with permission from Ref. [127]. Copyright 2021, ACS Applied Materials & Interfaces.

Gas sensors: The monitoring of harmful gases is of great significance to the protection of the ecological environment. In addition, with the prevalence of COVID-19, the sensors used to monitor exhaled gas are also very important for the prevention of respiratory diseases [116]. The integration of gas sensors with supercapacitors and energy-harvesters has also been widely studied [117,118,119,120,121,122,123]. For instance, Bao et al. [119] seamlessly integrated a solar cell, a rGO/CNT-based micro-supercapacitor, and a polypyrrole@rGO-based gas sensor on a flexible substrate using a continuous centrifugal coating strategy (Figure 7a). The supercapacitor exhibited an excellent volumetric capacitance (16.1 F cm^−3^) and a volumetric energy density (1.43 mWh cm^−3^). The system provided excellent gas detection performance toward NH_3_ and aniline.

Most self-powered gas sensors currently rely on the photovoltaic effect (PV), because UV light can activate the gas sensing ability of many metal oxides, and PV gas sensors can truly achieve the goal of zero power consumption for independent devices by harnessing ambient energy [124,125]. Storing PV energy can reduce excessive dependence on external energy sources. Antonio et al. [126] reported a perovskite (FMCPIB)-based device for selective detection of NO_2_. Its structure is shown in Figure 7b; photo-excited electrons and holes generated by FMCPIB were transferred to the FTO layer and the FMCPIB/carbon electrode contact interface, respectively, and stored. That was the mechanism of energy storage function. Additionally, the amine groups in FMCPB endowed it with a gas sensing capability. The results showed that the device could detect particle per million (ppm) concentrations of NO_2_ (detection limit with 1 V bias: 0.2 ppm) under light irradiation, and enable continuous operation for 1.7 h in darkness due to its energy-storage feature (Figure 7c). It can be seen that PV-based devices are very promising for both energy storage and gas sensing. The electrode potential involving the gas reactant changes with the gas concentration. According to this principle, a battery type gas sensor can be designed to reflect the detected gas concentration by its output voltage. So, Ho et al. [127] developed a battery–sensor hybrid that could be used as both a power supply and a gas sensor. The hybrid consists of carbon cloth deposited with zinc and Fe-N_x_ doped porous carbon catalyst, respectively, as electrodes. The sensing mechanism originated from the changes of electrode potential under varying NO_2_ concentrations (Figure 7d). The optimal sensitivity can be obtained by controlling the output current to adapt to the monitoring of different gas concentration ranges, such as 10 uA for 400–600 ppm, 10 or 100 uA for 600–800 ppm. In addition, NO_2_ was finally converted into NH_4_^+^, which meets the demands of green chemistry.

Biosensors: Biosensors can minimally and non-invasively detect various biological media (blood, sweat, urine, etc.) to obtain physiological information, such as blood glucose level, pH, [Na^+^] and [K^+^] content, etc. As mobile devices become ubiquitous, wearable integrated systems of energy-storage devices and biosensors provide a broad platform for personalized healthcare and will release the pressure of clinical resources in the future [110,111,128]. Several representative energy-storage devices integrated biosensing systems for sweat, interstitial fluid (ISF), and blood detection will be summarized below.

**Figure 8:**
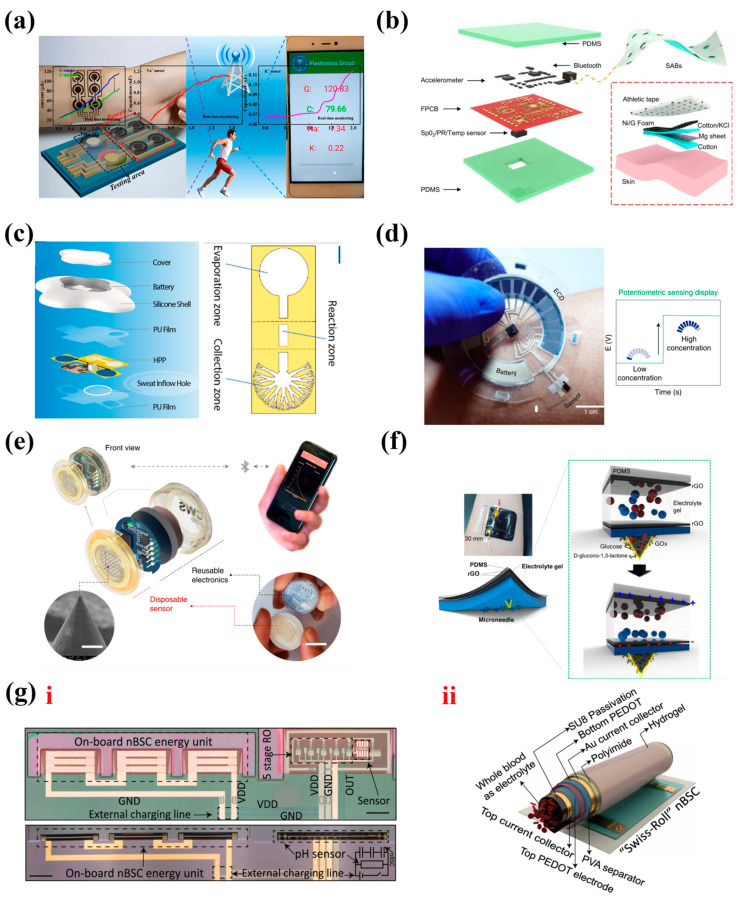
(**a**) Schematic of sensors that detect glucose, [Na^+^], and [K^+^] in sweat. Reprinted with permission from Ref. [129]. Copyright 2019, Nano Energy. (**b**) Schematic of FSAB-integrated multiple sensing system. Reprinted with permission from Ref. [130]. Copyright 2022, Nano Energy. (**c**) The exploded view of the system for monitoring of electrocardiogram signal and glucose in sweat. Reprinted with permission from Ref. [131]. Copyright 2022, Biosensors and Bioelectronics. (**d**) Schematic of sweat monitoring patch and the readout of potentiometric sensor changing with electrolyte concentration. Reprinted with permission from Ref. [132]. Copyright 2022, Nature Electronics. (**e**) The structure of highly integrated patch. Reprinted with permission from Ref. [136]. Copyright 2022, Nature Biomedical Engineering. (**f**) Schematic of self-charging supercapacitor for glucose sensing and its working principle. Reprinted with permission from Ref. [137]. Copyright 2022, ACS Applied Materials & Interfaces. (**g**) i. Schematic of nBSC; ii. microscope image of pH monitoring system before and after roll-up. Reprinted with permission from Ref. [141]. Copyright 2021, Nature Communications.

Shen et al. [129] integrated multiple micro-supercapacitors and sensors for detecting glucose, [Na^+^], and [K^+^] contents in human sweat on a single Poly (ethylene terephthalate) (PET) substrate (Figure 8a). The NiCo_2_O_4_-based micro-supercapacitors exhibited a high energy density of 0.64 μW cm^−2^ at the power density of 0.09 mW cm^−2^. Furthermore, the integrated system displayed excellent sweat detection performance with high sensitivities of 0.5 μA/μM for glucose, 0.031 nF/mM for [Na^+^], and 0.056 nF/mM for [K^+^]. In addition, it can be further integrated with wireless transmission technology, and realized with a real-time wireless detection of about 2 h though connecting with a smartphone. However, when no sweat is produced, special switch control is required to avoid invalid operation of the system. Yu et al. [130] reported a novel flexible sweat-activated battery (FSAB) with graphene/Ni foam and a Mg sheet as electrodes, respectively (Figure 8b), which provided a new idea to solve this problem. Once sweat was generated, it would be absorbed and diffused immediately by the bottom cotton layer of the battery, and the battery was then activated within 30 s. The FSAB exhibited a high energy capacity of 74.4 mA h and a power density of and 16.3 mW cm^−2^, which can power 120 LEDs for 4 h. In addition, they further developed a FSAB-integrated multiple sensing system for wireless and continuous physiological monitoring, including exercising intensity, skin temperature, pulse rate, and oxygen saturation in blood. However, these two systems are just embryonic forms, and their wearability is low due to the lack of appropriate packaging for their components. Ye et al. [131] developed a flexible hydrogel–paper patch, which could serve as low-impedance ECG electrodes due to the high conductivity, and the hydrophilic wettability endowed it with glucose-sensing performance (after the deposition of Pt nanoparticle), as shown in Figure 8c. A lithium battery was used to continuously supply energy for the device. The special packaging method made its structure more perfect and it could be directly attached to human skin. It is worth mentioning that a 3D microfluidic paper-based analytic device was designed to facilitate the collection, detection, and evaporation of sweat (Figure 8c), and avoided the accumulation of sweat and the pollution to the sensor, which is critical to the comfort and accuracy of sweat detection systems. In a recent work, a skin sweat sensing patch consisting of sensors, an Ag_2_O-Zn battery, an electrochromic display (ECD), and a small microcontroller unit (MCU) was developed (they were all stretchable except the MCU, Figure 8d) [132]. The working process was that the sensing electrodes detected sweat and generated voltage signals which were then processed by the MCU. Meanwhile, the battery powered the MCU and provided electrical potential to change the electrochromic pixel colors of the ECD and display them logically. Therefore, it could work independently without external wireless device connection. Based on this, the sensing platforms for detecting pH, Na^+^, glucose, and lactic acid were also established. The visual pixels array used in this study can quickly respond to physiological signals within 1 s, instead of directly using the electrical signal to display the change of the detection signals, the complex electrical components connection can be avoided, which is worth learning from.

ISF can provide more biochemical marker information than sweat because of its high degree of correlation with blood; further, the use of microneedle technology provides a painless ISF analysis method, instead of traditional painful blood collection [133,134,135]. Wang et al. [136] reported a fully integrated patch for wireless and continuous physiological monitoring, and featured small volume (just a coin size), easy attachment, and high sensitivity. As shown in Figure 8e, this patch consisted of a disposable microneedle array and reusable electronics. The microneedle array was endowed with sensing ability after corresponding enzyme modification, which displayed excellent real-time dynamic monitoring performance with sensing ranges and limits of 0–40 and 0.32 mM for glucose, 0–28 and 0.15 mM for lactate, 0–100 and 0.50 mM for alcohol, respectively. The reusable electronics were wirelessly connected to a smartphone application to realize data capture and visualization. A wireless rechargeable energy system (consisting of a charging coil and a lithium-ion battery) was engaged to power the electronics, and power optimizations enabled ~30 days of battery life. This patch is promising for use in actual human health monitoring. Interestingly, the organism itself is a huge energy source; collecting and storing the electric energy generated by redox reactions of biofluids provides a new idea for self-powered biosensing systems. For instance, Park et al. [137] assembled two pieces of PDMS coated with rGO and electrolyte (one side of which had a microneedle array), then loaded enzyme on the microneedle array, thus a self-charging supercapacitor with an excellent capacitance of 1600 F/g was prepared (Figure 8f). The glucose oxidase oxidizing glucose generated electrical energy to charge the supercapacitor, and the glucose concentration in the range of 10–11,000 μM could be reflected by the charging voltage. Meanwhile, a blood glucose alarm system was also established. Unfortunately, the integration degree of signal processing and transmission system and sensor is too low to be used in practice. This also reflects the urgent need for flexible integrated electronics.

In addition, microelectronics provides the possibility for in situ detection in blood. Several intravascular bioelectronic devices have been developed [138,139,140]. Schmidt et al. [141] reported a full microsystem-integrated nano-biosupercapacitor (nBSC) with a volume of 1 nL, which could generate a voltage of 1.6 V and display an average volumetric energy density of ~90 nWh mm^−3^ in blood (Figure 8g). Based on this, a self-powered system that consisted of three charged nBSCs, a nBSC based ring oscillator, and a pH sensor was successfully developed for blood monitoring, with a sensitivity of 5 ± 0.5 µF mm^−3^ per acidic pH and 2 ± 0.4 µF mm^−3^ per basic pH. Owing to the small volume, high electrochemical performance, and biocompatibility of nBSC, it provides more opportunities for the next generation of implantable microelectronics.

Although most of the current biosensor systems feature the characteristics of flexibility and biocompatibility, they are often unable to overcome the interference of various body fluids, limb movements, and the surrounding environment. Their comfort and stability are also relatively poor in the long-term wearing process. In addition, portable electronic devices for data capture and visualization are very scarce now, which is urgently needed to be overcome.

### 2.4. Multifunctional Integrated Type

At present, single function sensors have difficulty to meet the actual needs in practice, so systems with multi-sensing functions are the dominant direction of wearable electronics [142]. Li et al. [143] developed a system with a triple-mode sensor for pressure/temperature/light sensing and flexible micro-supercapacitors as the power supply. The triple-mode sensor exhibited excellent sensitivities of 19.3 kPa^−1^ for pressure, 0.0034 °C^−1^ for temperature, and fast UV response/recovery time of 0.8/0.9 s. Additionally, the micro-supercapacitor displayed a high volumetric capacitance of 148.25 F cm^−3^ and good cycle stability (93.75%, after 6000 cycles). Later, many similar multi-mode sensors have been developed [144,145,146,147,148,149], but the cross-talk between the measured parameters will lead to poor accuracy of single parameter measurements [118]. Therefore, the system integrated with multiple sensors shows advantages.

**Figure 9:**
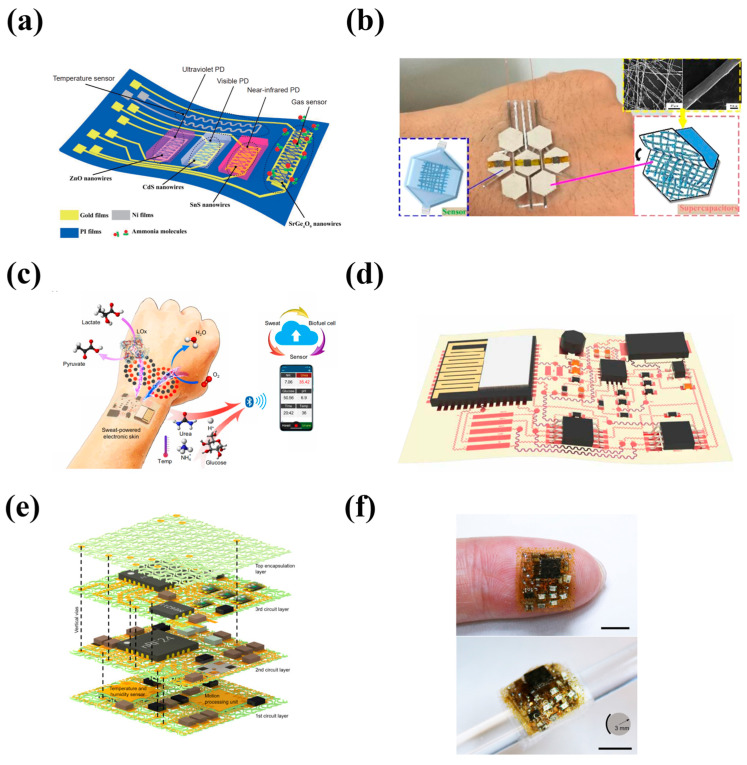
Schematic of (**a**) the system with multiple sensors. Reprinted with permission from Ref. [150]. Copyright 2020, Science China Materials. (**b**) Sensors (pressure sensors, photodetectors, and gas sensors) and on-chip micro supercapacitors integrated in one pixel. Reprinted with permission from Ref. [151]. Copyright 2017, Nano Energy. (**c**, **d**) The highly integrated soft E-skin with wireless multi-sensing. Reprinted with permission from Ref. [34]. Copyright 2020, Science Robotics. (**e**) Exploded view and (**f**) photographs of a highly integrated system based on stacked multilayer network materials. Reprinted with permission from Ref. [153]. Copyright 2022, Science Advances.

Figure 9a shows an example, a system with multiple sensors highly integrated on a flexible substrate [150]. Although the energy-storage devices were not integrated into it, the maturing of the printed circuit manufacturing process will clear the way for it. In an earlier study, Shen et al. [151] developed a multifunctional integrating system by only using rGO-on-PVDF-nanofibers for both sensing and energy storage, while sensors (pressure sensor, photodetector, and gas sensor) and on-chip micro-supercapacitors were integrated into a single pixel, as shown in Figure 9b. The rGO-on-PVDF-nanofibers exhibited a high capacitance of 595.4F/g, and four prepared supercapacitors in series supplied power for the sensor pixels, which could detect pulse, swallowing, voice, and body movement by the pressure sensor, light intensity by the photodetector, and the concentration of volatile organic compounds by the gas sensing part. This technology can be scaled-up to fabricate more integrated and higher performance multi-sensing systems in principle. As shown in Figure 9c, Gao et al. [34] demonstrated a highly integrated soft E-skin with wireless multi-sensing parts. This E-skin was powered by lactate biofuel cells, which featured high power density (3.5 mW cm^−2^) and long-term stability (continuous operation for 60 h) by use of unique zero- to three-dimensional nanomaterials. It could be used to detect various metabolic analytes (urea, NH_4_^+^, glucose, pH, etc.) and temperature, and could be further connected with tactile sensors for human–machine interfaces. All rigid components were highly integrated on a flexible substrate, and the serpentine-connected electrode arrays endowed the E-skin with the ability to withstand certain deformation (Figure 9d). The strategy of integrating rigid electronic components on flexible substrates provides a very promising solution for manufacturing flexible wearable electronics [5,152]. In a separate study, Zhang et al. [153] developed stacked multilayer network materials as a general framework for ultra-high integration of electronic components, which exhibited a moderate elastic stretchability of ~20% without elastic substrates, an ultra-high areal coverage of ~110%, and small size (11 mm by 10 mm). Based on this framework, as shown in Figure 9e,f, a multifunctional sensing integrated system powered by a lithium-ion battery was manufactured for physiological signals (relative humidity, temperature, and heart rate) wireless and real-time monitoring, and could work continuously for 80 min. At present, the flexibility of various electronic components (capacitor, resistor, potentiometer, transistor, relay, filter, etc.) is still in the initial stage of development. Compared with rigid components, their manufacturing is cumbersome and costly, which is not conducive to mass preparation, and the integration between flexible components is also greatly hindered. The “flexibility” strategy of rigid components in this study provides significant inspiration for the manufacture of small, stretchable, and complex electronic devices in the future.

## 3. Conclusions and Prospect

Wearable and flexible sensing systems have attracted extensive attention in the fields of personalized health monitoring, environmental monitoring, and human–machine interfaces due to the diversity of their performance and forms. In this review, we summarized the recent achievements of energy-storage-device-integrated sensing systems. It is easy to find that with the advance of materials science and process technology, we have made considerable achievements in this field. Features such as flexibility, extensibility, biocompatibility, comfort, and stability cannot be well expressed in a single system at the same time, it is a cutting-edge subject to gather all these advantages in one. In addition, the systems with energy-storage devices, especially multi-sensing systems with energy-harvesters and storage devices, can achieve continuous and stable wireless monitoring without external power supply, which is the major trend of the sensing field in the future.

Although considerable achievements have been achieved in energy-storage-device-integrated sensing systems, great challenges still remain for emerging and practical applications. These challenges include the following:

(1) There are still many issues to be solved for the energy-storage devices, as well as the electronics for energy management, multi-data capture, and visualization, such as flexibility, safety, stability, tedious manufacturing processes, and high costs.

(2) Interferences on sensing signals from the external environment (such as various pH condition, humidity, temperature) needs to be eliminated, but the research in this field is still relatively limited.

(3) The demand for device application scenarios is increasing, especially in some extreme conditions (such as extreme temperature and high pressure), which is a huge challenge for the integrated energy-storage and sensing field.

(4) In order to convert the real world into binary and transmit to intelligent terminals, the conversion between the sensing signals and the electrical signals needs to be extremely rigorous, stable, and accurate. However, at present, a single sensor has a large error, and the performance difference of the same batch of sensors is also large, which needs to be solved urgently.

(5) The comfort of wearable devices is also very important, but at present, most of them are not suitable for wearing for a long time, because they are air-proof and unfriendly to human skin. In addition, aesthetic design is also very important.

In the future, the development of new materials and ingenious structures may be a way to realize the miniaturization and flexibility of sensors, energy-storage devices, and various electronic components. Overall, wearable devices will greatly promote the development of the medical, environmental, robotic, and other fields.

## Figures and Tables

**Table 1:** Comparison of energy-storage devices and their integration modes for sensing systems.

Characteristics		Integration Mode		Devices	Mechanism	Characteristics	
Positives	Negatives					Positives	Negatives
Excellent sensing and energy-storage performance	Low integration degree	Component integration	Energy-storage devices for sensing systems	Supercapacitor	Electric double-layer capacitance or pseudocapacitance	High power, long cycle life	Low energy density
				Battery	Electrochemical redox reaction	High energy	Low power density
High integration degree	Sacrifice sensing and energy-storage performance	Function integration		Hybrid energy-storage device	Hybrid charge–storage mechanism	High energy density and high-power density	

Note: Component integration mode means that energy storage device and sensors are integrated as independent units; function integration mode means that the same device has both sensing and energy-storage functions.

## Data Availability

Suggested Data Availability Statements are available in section “MDPI Research Data Policies” at https://www.mdpi.com/ethics.
